# Impact of novel palmitoylated prolactin-releasing peptide analogs on metabolic changes in mice with diet-induced obesity

**DOI:** 10.1371/journal.pone.0183449

**Published:** 2017-08-18

**Authors:** Veronika Pražienková, Martina Holubová, Helena Pelantová, Martina Bugáňová, Zdenko Pirník, Barbora Mikulášková, Andrea Popelová, Miroslava Blechová, Martin Haluzík, Blanka Železná, Marek Kuzma, Jaroslav Kuneš, Lenka Maletínská

**Affiliations:** 1 Institute of Organic Chemistry and Biochemistry, Academy of Sciences of the Czech Republic, Prague, Czech Republic; 2 Institute of Microbiology, Academy of Sciences of the Czech Republic, Prague, Czech Republic; 3 Faculty of Chemical Technology, University of Chemistry and Technology Prague, Prague, Czech Republic; 4 Institute of Experimental Endocrinology, Biomedical Research Center, Slovak Academy of Sciences, Bratislava, Slovak Republic; 5 Department of Human and Clinical Pharmacology, University of Veterinary Medicine, Košice, Slovak Republic; 6 Institute of Physiology, Academy of Sciences of the Czech Republic, Prague, Czech Republic; 7 Institute of Medical Biochemistry and Laboratory Diagnostics, Charles University in Prague and General University Hospital, Prague, Czech Republic; 8 Centre of Experimental Medicine, Institute for Clinical and Experimental Medicine, Prague, Czech Republic; 9 Diabetes Centre, Institute for Clinical and Experimental Medicine, Prague, Czech Republic; University of Melbourne, AUSTRALIA

## Abstract

Analogs of anorexigenic neuropeptides, such as prolactin-releasing peptide (PrRP), have a potential as new anti-obesity drugs. In our previous study, palmitic acid attached to the N-terminus of PrRP enabled its central anorexigenic effects after peripheral administration. In this study, two linkers, γ-glutamic acid at Lys^11^ and a short, modified polyethylene glycol at the N-terminal Ser and/or Lys^11^, were applied for the palmitoylation of PrRP31 to improve its bioavailability. These analogs had a high affinity and activation ability to the PrRP receptor GPR10 and the neuropeptide FF2 receptor, as well as short-term anorexigenic effect similar to PrRP palmitoylated at the N-terminus. Two-week treatment with analogs that were palmitoylated through linkers to Lys^11^ (analogs 1 and 2), but not with analog modified both at the N-terminus and Lys^11^ (analog 3) decreased body and liver weights, insulin, leptin, triglyceride, cholesterol and free fatty acid plasma levels in a mouse model of diet-induced obesity. Moreover, the expression of uncoupling protein-1 was increased in brown fat suggesting an increase in energy expenditure. In addition, treatment with analogs 1 and 2 but not analog 3 significantly decreased urinary concentrations of 1-methylnicotinamide and its oxidation products N-methyl-2-pyridone-5-carboxamide and N-methyl-4-pyridone-3-carboxamide, as shown by NMR-based metabolomics. This observation confirmed the previously reported increase in nicotinamide derivatives in obesity and type 2 diabetes mellitus and the effectiveness of analogs 1 and 2 in the treatment of these disorders.

## Introduction

The identification of new substances with targeted anti-obesity potencies is needed, as several anti-obesity drugs, namely derivatives of neurotransmitters, have been withdrawn from the market because of significant side effects. Analogs of anorexigenic peptides seem to be a better alternative for the development of new anti-obesity drugs. Liraglutide, a glucagon-like peptide-1 (GLP-1) analog acylated with palmitic acid that was originally developed as a type-2 diabetes mellitus (T2DM) drug, has recently been approved in the U.S.A for obesity treatment (*Saxenda*). In addition to GLP-1, pancreatic polypeptide (PP) and peptide YY (PYY)—peptides of gut origin affecting the gastrointestinal tract—have central anorexigenic effects (gut-brain peptides) and have been evaluated as possible treatments for obesity. Several brain anorexigenic neuropeptides such as α-melanocyte stimulating hormone (α-MSH), cocaine- and amphetamine-regulated transcript (CART) peptide or prolactin releasing peptide (PrRP) originate in the brain where they also have anorexic actions. An α-MSH analog modified with a fatty acid has been shown to be a stable substance with strong anorexigenic effect [1]. However, its adverse effects on skin led to the termination of further research [2]. Furthermore, the CART peptide receptor has not yet been identified, which makes it pharmacologically less desired [3].

PrRP seems to be a good candidate for anti-obesity drug development. Its anorexigenic effect after central administration has been proven [4], while its prolactin-secreting properties were not confirmed by later studies [5, 6]. However, the original name remained unchanged. PrRP binds with a similar affinity to its receptor GPR10 and also to another G-protein coupled receptor NPFF2, whose primary endogenous ligand is neuropeptide FF [7]. Mice with an inactivated PrRP gene or GPR10 receptor are obese, which supports the anorexigenic properties of PrRP [8, 9].

According to the results of our recent study, peripheral administration of PrRP modified by an addition of myristoyl or palmitoyl acid showed similar central anorexigenic effect as PrRP. Furthermore, its long-lasting and sustained anorexigenic properties had beneficial effect on obesity-related metabolic disturbances, and it exhibited prolonged stability in blood [10]. Consequently, palm-PrRP31 was used as the basis for the further development of anorexigenic substances. Moreover, analogs of palm-PrRP in which the C-terminal Phe was replaced with a non-coded aromatic acid showed high binding affinity for both GPR10 and NPFF2, as well as anorexigenic properties that were similar to the palm-PrRP31 consisting of natural amino acids [11].

Metabolomics, which focuses on the characterization of all significant metabolites present in a biological sample, can provide insight into the mechanisms of different pathological processes as well as the therapeutic actions [12]. Therefore, it may serve as a useful tool for the assessment of the effects of different therapeutic interventions [13, 14]. A NMR-based metabolomic approach was also successfully applied in our previous study exploring the effects of treatment with metformin, vildagliptin and a combination of both in a mouse model of diet-induced obesity (DIO) [15].

In this study, we aimed to explore the biological effects of several new lipidized PrRP analogs with improved solubility and bioavailability due to changes in the position of the fatty acid attached to the peptide. PrRP was acylated with palmitic acid through a linker that was situated at amino acid 11, originally Arg, modified for Lys, as well both position 11 and at the N-terminus. We examined whether the location of the fatty acid(s) affected the affinity for the receptor and the anorexigenic effect of the acylated PrRP, as well as the effects of these analogs on food intake, body weight (BW) and metabolic parameters in DIO mice after repeated peripheral administration. Moreover, NMR-based metabolomics of mouse urine was applied to evaluate the complex responses to these interventions.

## Material and methods

### Peptide synthesis and iodination

Human PrRP analogs (see Table 1 for structures) and 1DMe (D-YL(N-Me)FQPQRF-NH_2_), a stable analog of NPFF, were synthesized and purified as described previously [10] using Fmoc strategy. The peptide sequences were assembled in a solid-phase synthesizer Liberty Blue (CEM, Mathews, NC, USA) by stepwise coupling of the corresponding Fmoc-amino acids to the growing chain on TENTA GEL S RAM resin (200–400 mesh, 0.25 mmol/g) (IRIS, Biotech GmbH, Marktredwitz, Germany). Fully protected peptide resins were synthesized according to a standard procedure involving (i) cleavage of the N^α^- Fmoc protecting group with 20% piperidine in dimethylformamide (DMF), (ii) coupling, mediated by mixtures of coupling reagents diisopropylcarbodiimide **(**DIC)/Oxyma in DMF. Lipidization of the PrRP analogs was performed as shown in [16] on fully protected peptides on resine after the coupling of γ-Glu or 1,13-diamino-4,7,10-trioxatridecan-succinamic acid (TTDS) as the last step. For Lys^11^, special protecting group of side-chain, N-[1-(4,4-dimethyl-2,6-dioxocyclohex-1-ylidene)ethyl] (Dde) was used. Cleavage of Dde was performed by 2% hydrazine monohydrate in *N*-Methyl-2-pyrrolidone.

**Table 1:** Structure of human PrRP31 and its analogs.

Analog	Sequence
**human PrRP31**	SRTHRHSMEI**R**TPDINPAWYASRGIRPVGRF-NH_2_
**analog 1**	SRTHRHSMEI **K** (N-γ-E (N-palm)) TPDINPAWYASRGIRPVGRF-NH_2_
**analog 2**	SRTHRHSMEI **K** (N-TTDS(N-palm)) TPDINPAWYASRGIRPVGRF-NH_2_
**analog 3**	(N-TTDS(N-palm)) S RTHRHS MEI **K** (N-TTDS(N-palm)) TPDINPAWYASRGIRPVGRF-NH_2_

TTDS—1,13-diamino-4,7,10-trioxatridecan-succinamic acid

On completion of syntheses, the deprotection and detachment of peptides from the resins were carried out simultaneously, using a trifluoroacetic acid (TFA)/H_2_O/Triisopropylsilane (TIS) (95:2.5:2.5) cleaving mixture. Each of the resins was washed with a dichloromethane and the combined TFA filtrates were evaporated at room temperature. The precipitated residues were triturated with tert-butyl-methylether, collected by suction and dried by lyophilization. The peptides were purified by HPLC using a Waters instrument with Delta 600 pump, 2489 UV/VIS detector (Milford, MA, USA).

The purity and identity of all of the peptides were determined by analytical HPLC and by using a MALDI-TOF/TOF mass spectrometer (Bruker Daltonics, Germany) (see S1 Table).

Molecular weight was determined by MALDI MS technique (Bruker Daltonics, Germany). In HPLC analyses, retention time in minutes, separation on 25 x 0.46 cm column, 5 μm (Vydac 218TP C18, Separations Group, Hesperia, USA), Waters Alliance instrument, detection at 220 nm. Gradient 2–80% of acetonitrile in 0.1% aqueous TFA, 25 min, 80–100% 2 min, flow 1ml/min.

Human PrRP31 and 1DMe were iodinated at Tyr^20^ and D-Tyr^1^, respectively, with Na^125^I (Izotop, Budapest, Hungary) as described previously [16].

### Binding to intact plated cells and cell membranes

CHO-K1 cells expressing the GPR10 receptor (Thermo Fisher Scientific Inc., Waltham, MA, USA) were grown according to the manufacturer's instructions. Saturation and competitive binding experiments were performed according to the methods of Motulsky and Neubig [17]. CHO-K1 cells were incubated with 0.5–5 nM ^125^I-hPrRP31 in the saturation experiments or with 0.03 nM ^125^I-hPrRP31 and 10^−11^–10^−5^ M non-radioactive ligands in the competitive binding experiments. The experiments were performed on plated cells that were incubated for 60 min at 25°C. Non-specific binding was determined using 10^−5^ M PrRP31. The binding assays involving human NPFF2 receptor membranes obtained from Perkin Elmer were performed as described in [18].

### Beta-lactamase-dependent FRET assay

CHO-K1 cells overexpressing GPR10 were plated at 40,000 cells/well in 128 μl of assay medium (DMEM containing 10% dialyzed FBS, 0.1 mM NEAA, 25 mM HEPES, 1% penicillin/streptomycin, and 2 mM L-glutamine) in a 96-well plate and were incubated for 20 h at 37°C/5% CO_2_. The assay was performed according to the manufacturer's protocol [11]. The plates were read using a Tecan Infinite M1000 fluorescent plate reader (Tecan Group Ltd., Männedorf, Switzerland) with a 405 nm excitation wavelength and a 460 nm or 530 nm emission wavelength via bottom read.

### Acute food intake in fasted lean mice

All of the animal experiments followed the ethical guidelines for animal experiments and the Act of the Czech Republic Nr. 246/1992 and were approved by the Committee for Experiments with Laboratory Animals of the Academy of Sciences of the Czech Republic.

Male C57BL/6 mice from Charles Rivers Laboratories (Sulzfeld, Germany) were housed at a temperature of 23°C with a daily 12 h light/dark cycle (lights on at 6:00 am). The mice were given *ad libitum* water and a standard rodent chow diet Ssniff^®^ R/M-H (Ssniff Spezialdiäten GmbH, Soest, Germany) and housed until three months of age. The mice were fasted overnight (17 h) prior to the food intake experiment and then subcutaneously (SC) injected with 200 μl of either saline or PrRP analogs at doses of 5 mg/kg (all dissolved in saline, n = 5–6). Fifteen minutes after injection, the mice were given weighed food pellets. The pellets were weighed every 30 min for at least 6 h, and the animals had free access to water during the experiment. The results are expressed as grams of food consumed.

### Fos immunohistochemistry

For c-Fos immunohistochemical processing, male mice with free access to water that had been fasted overnight (n = 4) were SC injected with saline (Sal), or analog 1 at a dose of 5 mg/kg. Ninety minutes after injection, the mice were deeply anesthetized with sodium pentobarbital (50 mg/kg, intraperitoneally) and perfused transcardially. The brains were withdrawn, and c-Fos immunoreactivity was determined as described in [19, 20].

For the immunohistochemical study, the c-Fos rabbit monoclonal antibody (Cell Signaling Technology, #2250S) detecting total level of endogenous c-Fos protein was used in final dilution (1:2000) for 48 h (4°C).

### Long-term effects of lipidized PrRP analogs on body weights and biochemical and metabolic parameters in mice with diet-induced obesity

#### Animals, diets and treatment

Inbred C57BL/6 male mice that were 3 weeks old were obtained from Charles River Laboratories (Sulzfeld, Germany). The mice were housed under controlled conditions at a constant temperature of 22 ± 2°C, a relative humidity of 45–65% and a fixed daylight cycle (6 am– 6 pm), with 5 mice per cage. The animals were provided free access to water and the standard rodent chow diet Ssniff^®^ R/M-H (Ssniff Spezialdiäten GmbH, Soest, Germany) containing 33%, 9% and 58% of calories from proteins, fats and carbohydrates, respectively.

From 8 weeks of age, the mice were fed in house made high-fat (HF) diet to induce obesity. The energy content of the HF diet was 5.3 kcal/g, with 13%, 60% and 27% of the calories derived from proteins, fats and carbohydrates, respectively. The diet was composed of 40% standard chow, 34% powdered cow-milk-based human baby formula, 25% lard, and 1% corn starch w/w [10]. After twelve weeks of HF-diet feeding, two weeks of SC administration of lipidized PrRP analogs was initiated while the mice were kept on the HF diet. The following experimental groups were established: A. saline, B. 5 mg/kg of analog 1, C. 5 mg/kg of analog 2, and D. 5 mg/kg of analog 3 (n = 10). The compounds were dissolved in saline and administered subcutaneously (into abdominal part) twice a day at a dosing volume of 0.15 ml. Food intake and BW were monitored daily during the dosing period.

#### Urine collection for NMR-based metabolomics

Urine samples for NMR-based metabolomics were collected prior to the start of the interventions and at the end of the experiment. For overnight urine collection (from 5 pm to 8 am), the mice were housed in individual metabolic cages (Tecniplast, Buguggiate, Italy) without access to food. Samples were collected with the addition of NaN_3_ and stored at -80°C until NMR analysis.

#### Blood sampling for biochemical analyses and tissue dissection

At the end of the experiment, the mice were sacrificed by decapitation. The trunk blood was collected, and the blood plasma was separated and stored at -20°C. The visceral adipose tissues (VAT) (from abdominal part), subcutaneous adipose tissues (SCAT) (all adipose tissue depots under the skin), perirenal adipose tissues, interscapular brown adipose tissues (BAT), and livers of all of the mice were dissected, weighed, flash-frozen in liquid nitrogen, and stored at -70°C for later extraction of RNA.

#### Determination of hormonal and biochemical parameters

The plasma insulin concentrations were measured using an RIA assay (Millipore, St. Charles, MI, USA), and the leptin concentrations were determined by ELISA (Millipore, St. Charles, MI, USA). The blood glucose levels were measured using a Glucocard glucometer (Arkray, Kyoto, Japan). The plasma triglyceride levels were measured using a quantitative enzymatic reaction (Sigma, St. Louis, MO, USA), and the free fatty acids (FFA) levels were determined using a colorimetric assay (Roche, Mannheim, Germany). Cholesterol was determined by colorimetric assay (Erba Lachema, Brno, Czech Republic). All measurements were performed according to the manufacturer's instructions.

Rate of insulin resistance was expressed by homeostatic model assessment (HOMA) calculated as (fasting glucose level, mmol/l) x (fasting insulin level, pmol/l) divided by 22.5 [21].

#### Determination of mRNA expression

The mRNA expression of the genes of interest was determined in samples from the mice treated with analogs 1 and 2 only. Samples of adipose tissues (subcutaneous, visceral, and brown) and livers were processed as described in [22]. The mRNA expression of the genes of interest (*Fasn*, *Lpl*, *Lep*, and *Adipoq* in SCAT and VAT; *Fasn*, *Lpl*, *Pck1*, *Srebf*, *Cpt1a* and *Cpt1b* in liver; *Ucp1* in BAT) was determined using an ABI PRISM 7500 instrument (Applied Biosystems, Foster City, CA, USA). The expression of beta-2-microglobulin (*B2m*) was determined for adjustments for variations in the amounts of input RNA and normalization of the efficiency of reverse transcription (*Fasn*–Fatty Acid Synthase, *Lpl*–Lipoprotein Lipase, *Lep*–Leptin, *Adipoq*–adiponectin, *Pck1* –Phosphoenolpyruvate Carboxykinase 1, *Srebf*–Sterol Regulatory Element-Binding transcription Factor, *CPT1a/b*–Carnitine Palmitoyltransferase 1a/b, and *Ucp1* –Uncoupling Protein 1).

### Analysis of binding data and statistics

Data are presented as the means ± SEM. The competitive binding curves were plotted using GraphPad software (San Diego, CA, USA), and the best fits were compared for single-binding-site models (IC_50_ values were obtained from nonlinear regression analysis). Inhibition constants (K_i_) were calculated from the IC_50_ values using the Cheng-Prusoff equation [23]. The concentration of the radioligand was 0.1 nM for cell membranes of CHO-K1 cells overexpressing NPFF2 and 0.03 nM for CHO-K1 cells overexpressing GPR10. The K_d_ calculated from the saturation experiments was 1.06 ± 0.36 nM for CHO-K1 cells with GPR10 and 0.72 ± 0.12 nM for membranes with NPFF2 [10].

The results from the lactamase assay were analyzed using nonlinear regression by log agonist vs. response using GraphPad software. EC_50_ was calculated as the concentration of the peptide that yielded 50% of the maximal effect.

Statistical analysis of the DIO model was performed using unpaired t-tests or repeated measures ANOVA with a Bonferroni's *post hoc* test as indicated in the figure and table legends. The differences between the control and treated groups were considered significant at P < 0.05. Where error bars are not visible in the figures, the standard error was within the symbol size.

### NMR-based metabolomics

#### NMR experiments

The urine samples were thawed at room temperature and centrifuged at 13 684 x g for 5 min prior to the NMR experiments. A 200-μl aliquot of supernatant was diluted with 340 μl of H_2_O mixed with 60 μl of phosphate buffer (1.5 M KH_2_PO_4_ in D_2_O containing 2 mM NaN_3_ and 0.1% trimethylsilyl propionic acid (TSP), pH 7.4) and transferred to a 5-mm NMR tube. The NMR data were acquired with a 600 MHz Bruker Avance III spectrometer (Bruker BioSpin, Rheinstetten, Germany) equipped with a 5-mm TCI cryogenic probe head. All of the experiments were performed at 300 K. Automatic tuning, matching, shimming and adjusting of the 90° pulse length were performed for each sample.

As mouse urine naturally contains urinary proteins, the NMR spectra were acquired using a Carr-Purcell-Meiboom-Gill (CPMG) pulse sequence with water presaturation (Bruker pulse sequence cpmgpr1d) to eliminate the strong protein background [24]. The following parameters were applied: presaturation during relaxation delay (4 s) using a 25-Hz saturation pulse centered on water resonance; number of scans (NS) = 64; number of data points (TD) = 64k; spectral width (SW) = 20 ppm; echo time = 0.3 ms; and loop for T2 filter = 126. For better metabolite identification, a short J-resolved experiment with presaturation (NS = 2, SW = 16 ppm, TD = 8k, number of increments = 40, SW = 78.125 Hz in the indirect dimension, and relaxation delay = 2 s) was executed for all samples. Additional two-dimensional ^1^H-^1^H correlation spectroscopy (COSY) and ^1^H-^13^C heteronuclear multiple-quantum correlation (HMQC) experiments (Bruker pulse sequences cosygpprqf and hmqcphpr) were performed for selected samples.

The acquired data were processed using Topspin 3.2 software (Bruker BioSpin, Rheinstetten, Germany). Line broadening of 0.3 Hz was applied on free induction decays (FIDs) prior to Fourier transformation. The spectra were automatically phased, baseline corrected and referenced to the signal of TSP (0.00 ppm).

#### Evaluation of NMR data

The assignment of individual metabolites was achieved by comparing the proton spectra with the spectra of pure metabolites using Chenomx NMR Suite 7.6 (Chenomx Inc., Edmonton, AB, Canada), the HMDB database and previously published metabolomic data [25, 26]. The identification of metabolites was supported through J-resolved and COSY experiments and, if possible, confirmed using carbon chemical shifts extracted from the HMQC spectra.

For quantitation of individual metabolites, the CPMG spectra were uniformly binned in the range of 0.10–10.00 ppm to 0.01-ppm intervals using AMIX software (Bruker BioSpin, Rheinstetten, Germany). This procedure reduced the dimensionality of the data, eliminated chemical shift variations and thus simplified the reproducible automatic quantification. Regions corresponding to water (4.60–4.90 ppm) and urea (5.60–6.00 ppm) were removed prior to analysis. As the dilutions of the original urine samples differed (due to the differences in water intake during urine collection), all of the spectra were normalized to the total spectral area (TSA). For each identified metabolite, one representative signal was chosen and then quantified as the sum of appropriate adjacent 0.01-ppm bins to cover the whole signal width. The metabolite concentration was expressed as the TSA-normalized intensity of the respective signal. For selection of an appropriate statistical method (parametric or non-parametric) the Lilliefors test of normality was performed. Because its results showed a normal distribution of the vast majority of data, normalized signal intensities were then subjected to the unequal variance unpaired t-test to determine the statistical significance of the detected changes in metabolite levels. Metabolite alterations with a P < 0.05 were considered statistically significant. Statistical analysis was performed using Matlab software (“MATLAB version 8.6 (R2015b). Natick, Massachusetts: The MathWorks Inc., 2015.,” n.d.).

Finally, the intensities of the significantly altered metabolites were subjected to Pearson's correlation with the set of biometric and metabolic parameters acquired at the end of the experiment. Statistically significant correlations (P < 0.05) in which the absolute value of the correlation coefficient was higher than 0.5 were indicative of a potential relationship between that particular metabolite and obesity.

## Results

### Peptide synthesis and iodination

The structures of PrRP31 and its analogs that were used in this study are shown in Table 1. All of the peptide sequences were assembled on solid phase support, and the purity of the peptides was higher than 95%. Three palmitoylated analogs of PrRP31 were prepared by the attachment of palmitic acid through a linker (γ-glutamic acid (analog 1) or 1,13-diamino-4,7,10-trioxatridecan-succinamic acid (TTDS) (analog 2)) to the side chain amino group of Lys^11^. In addition, analog 3 has a second attached palmitic acid through the TTDS linker at the N-terminal Ser.

Rat PrRP31, human PrRP31 and 1DMe were iodinated at Tyr^20^ and D-Tyr^1^, respectively, using IODO-GEN^TM^ as described previously [27].

### Competitive binding and beta-lactamase assays of PrRP analogs

The human PrRP31 or lipidized PrRP analogs competed with human ^125^I-PrRP for binding to CHO-K1 cells overexpressing human GPR10 or NPFF2 with a K_i_ in the nanomolar range, as shown in Table 2 and S1 Data. The K_i_ value for the stable analog 1DMe in the competitive binding assay using ^125^I-1DMe and membranes of CHO-K1 cells overexpressing human NPFF2 was 1.80 ± 0.54 nM. The PrRP analogs with fatty acids showed an affinity for GPR10 that was comparable to the natural peptide PrRP31. The lipidized PrRP analogs competed with ^125^I-1DMe and had a similar Ki to the unlabeled 1DMe. Thus, palmitoylated PrRP31 showed a very high affinity for both the GPR10 and NPFF2 receptors.

**Table 2:** Biological properties of PrRP31 analogs.

Analog	^125^I-human PrRP31 binding to human GPR10	% binding human PrRP31	^125^I-1DMe binding to human NPFF2	% binding human PrRP31	Activation of *bla* reporter gene in GPR10 overexpressing cells	Food intake in fasted mice (5mg/kg SC)
	K_i_ [nM]		K_i_ [nM]		EC_50_ [pM]	[% saline-treated group (45min)]
**saline**						100.0 ± 4.5
**human PrRP31**	4.58 ± 0.66	100	50.0 ± 24.3	100	243 ± 50	96.3 ± 8.5
**analog 1**	5.01 ± 1.03	91	39.1 ± 23.1	128	46 ± 12	14.2 ± 3.3
**analog 2**	3.79 ± 0.47	121	6.6 ± 3.3	761	83 ± 18	15.2 ± 5.4
**analog 3**	32.40 ± 3.57	14	8.6 ± 1.9	580	284 ± 54	27.6 ± 6.5

In competitive binding, Ki was calculated using Cheng-Prusoff equation. EC_50_ is the concentration of peptide at 50% of maximal effect. Food intake was analyzed at 45 min after SC injection of an analog when the maximal effect is observed and is expressed in percentage of saline-treated group consumption. The data represent the means ± SEM of at least three separate experiments.

Activation of the beta-lactamase reporter gene by natural PrRP31 or PrRP analogs in CHO-K1 cells overexpressing GPR10 revealed the agonistic characteristics of the PrRP analogs, with an EC_50_ in the sub-nanomolar range (Table 2, S1 Data). Similar to the results of the binding assays, we found that the lipidized analogs were more effective at activating GPR10 than the natural peptide.

### Acute food intake in fasted lean mice

The results of the food intake experiments after SC injection of the fasted lean mice at the time of the highest effect (45 min after injection) are shown in Table 2 and S1 Data. As expected, the food intake of the mice SC injected with natural PrRP31 was not affected (Table 2, S1 Data). However, in accordance with the high affinity for the GPR10 and NPFF2 receptors observed in the binding assay (Table 2, S1 Data), the palmitoylated PrRP31 analogs significantly decreased food intake. Analogs 1 and 2 had stronger anorexigenic effects compared to analog 3 (Table 2, S1 Data).

### Fos immunohistochemistry

**Fig 1:**
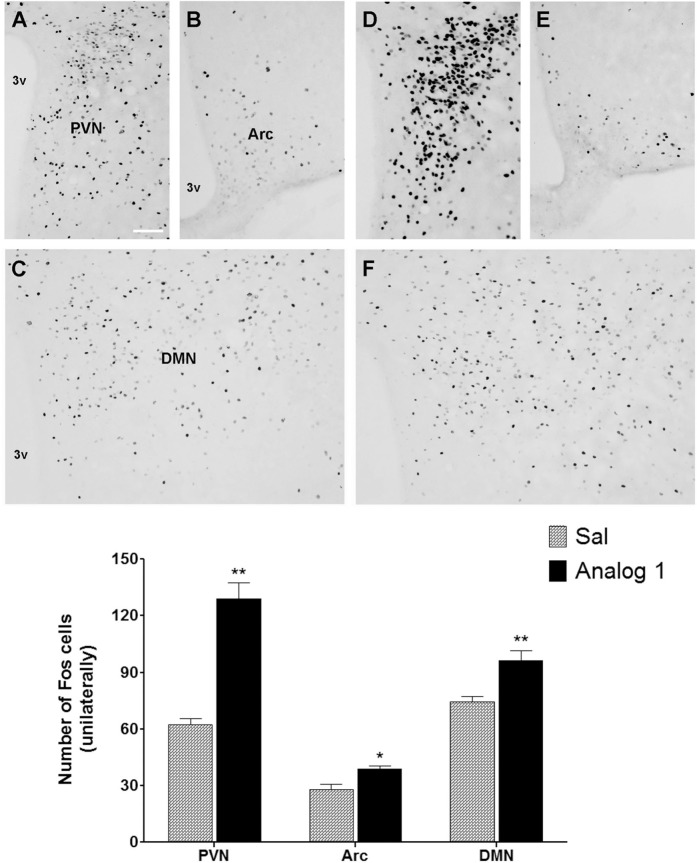
Effect of analog 1 on neuronal activity in food intake-regulating areas in mouse brain. The representative photographs of Fos immunostained cells in coronal section of PVN (A, D), Arc (B, E) and DMN (C, F) ninety minutes after SC application of saline (A-C) and analog 1 in dose of 5 mg/kg (D-F) in overnight fasted mice. The graph represents the means of Fos-immunostained cells in coronal sections (n = 3 sections per mice) of PVN, Arc and DMN analyzed via one-way ANOVA followed by Tukey´s HSD post-hoc test and expressed as mean ± S.E.M. (n = 4 mice per group). * P ˂ 0.05 and ** P ˂ 0.01 vs Sal. The scale bar represents 50 μm. PVN–paraventricular hypothalamic nucleus, Arc–arcuate hypothalamic nucleus, DMN–dorsomedial hypothalamic nucleus, 3v –third brain ventricle, Sal–saline.

The central effects of peripherally administered analog 1 were confirmed by a significant increase in c-Fos immunoreactivity in the hypothalamic arcuate nucleus (Arc), the paraventricular nucleus (PVN) and dorsomedial (DMN) nucleus, which are involved in the regulation of food intake (Fig 1).

### Long-term effects of lipidized PrRP analogs on body weights and biochemical and metabolic parameters in DIO mice

#### Impact of the treatments on food intake, body weight and body composition of DIO mice

**Fig 2:**
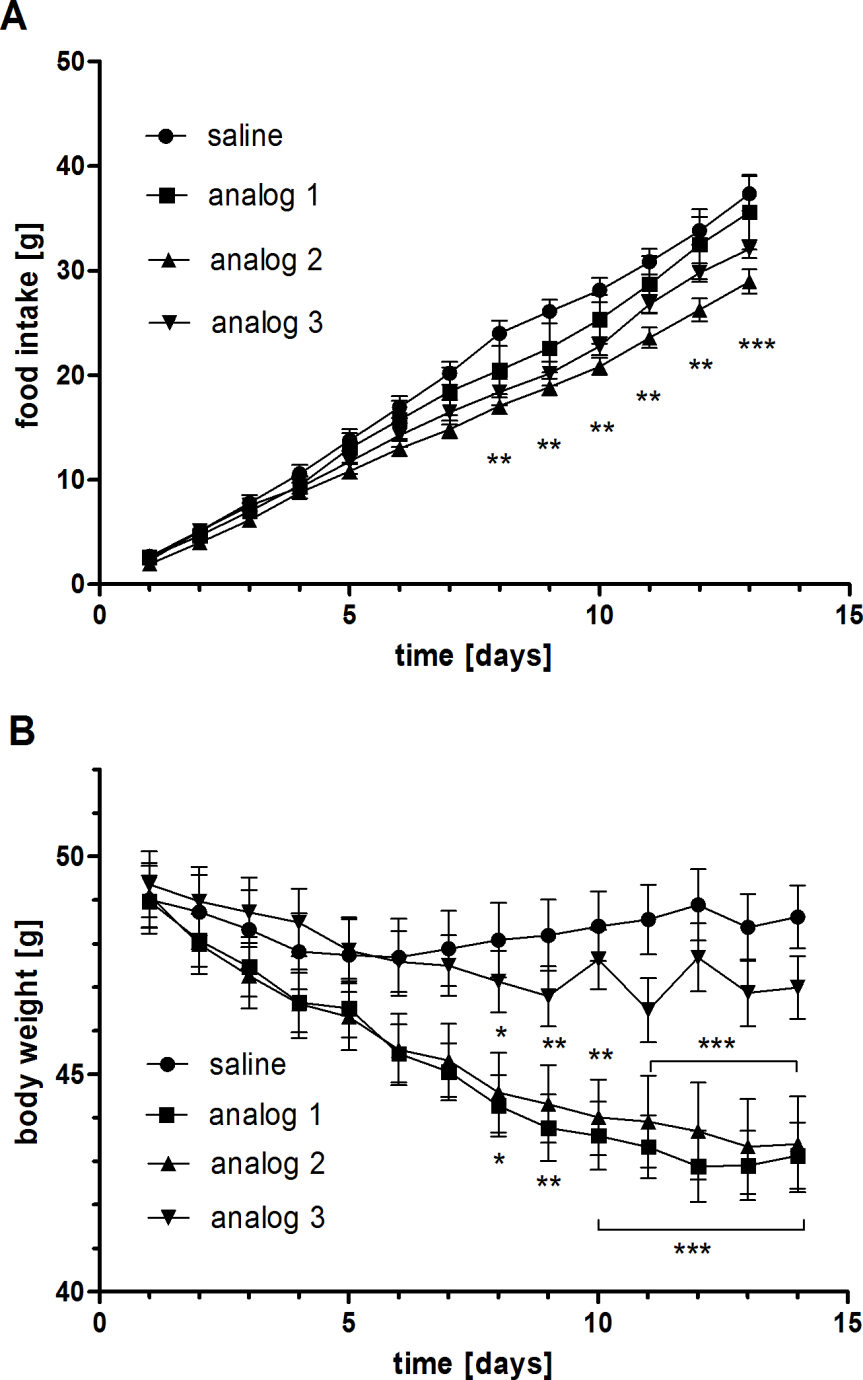
**Cumulative food intake (A) and body weights (B) of DIO mice during the treatment with lipidized PrRP analogs.** The data are presented as the mean ± SEM. Statistical analysis was performed using the repeated measures ANOVA with the Bonferroni´s post hoc test. * P < 0.05, ** P < 0.01, *** P < 0.001 vs. the HF diet-fed group treated with saline.

Two weeks of SC treatment with PrRP analog 2 significantly reduced food intake compared to the saline-treated control group (Fig 2A, S1 Data). Although the reduction in food intake after treatment with analog 1 did not reach statistical significance, the BW-reducing effects of the compound were surprisingly comparable to analog 2 (Fig 2B, S1 Data). At the end of the experiment, the average BW of the mice treated with analogs 1 and 2 decreased by 12% and 11.75%, respectively. Analog 3 did not have any significant effects on food intake or BW, but there was a trend toward a decrease in both parameters.

Treatment with analog 1 significantly reduced the weights of the SCAT and perirenal adipose tissue, and administration of analog 2 reduced the weight of the perirenal adipose tissue. Importantly, both compounds reduced the liver weights. Analog 3 did not have any significant effects on body composition in the DIO mice (Table 3, S1 Data).

**Table 3:** Adipose tissue and liver weights in DIO mice at the end of the experiment.

Treatment	SCAT [% of BW]	VAT [% of BW]	Perirenal AT [% of BW]	Total AT [% of BW]	Liver [g]
**saline**	8.00 ± 0.24	4.15 ± 0.37	3.26 ± 0.13	15.85 ± 1.65	1.60 ± 0.10
**analog 1**	7.06 ± 0.29 *	4.37 ± 0.28	2.72 ± 0.11 **	14.59 ± 0.32	1.27 ± 0.05 **
**analog 2**	8.33 ± 0.42	4.29 ± 0.36	2.73 ± 0.17 *	15.70 ± 0.53	1.31 ± 0.06 *
**analog 3**	7.85 ± 0.39	4.74 ± 0.29	3.09 ± 0.16	16.13 ± 0.38	1.58 ± 0.09

The data are presented as the mean ± SEM. Statistical analysis was performed using the unpaired t-test.

* P < 0.05

** P < 0.01 vs. the control group treated with saline. SCAT–subcutaneous adipose tissue, VAT–visceral adipose tissue.

#### Impact of the treatments on the metabolic parameters in the blood of DIO mice

All of the lipidized PrRP analogs tended to decrease fasting glucose levels after long-term SC administration. Analogs 1 and 2 significantly decreased plasma levels of insulin, HOMA index, triglycerides, cholesterol, free fatty acids and leptin (Table 4, S1 Data).

**Table 4:** Metabolic parameters in blood of DIO mice at the end of the experiment.

Treatment	Glucose [mmol/l]	Insulin [ng/ml]	HOMA index	FFA [mmol/l]	Triglycerides [mg/ml]	T Chol [mmol/l]	Leptin [ng/ml]
**saline**	8.22 ± 0.59	3.42 ± 0.26	215.53 ± 1.18	0.69 ± 0.04	0.66 ± 0.03	4.90 ± 0.13	44.42 ± 2.67
**analog 1**	7.12 ± 0.36	1.57 ± 0.14 ***	85.70 ± 0.39 ***	0.54 ± 0.03 **	0.44 ± 0.03 ***	4.26 ± 0.22 *	22.67 ± 2.72 ***
**analog 2**	7.26 ± 0.31	1.55 ± 0.27 ***	86.27 ± 0.64 ***	0.52 ± 0.02 **	0.44 ± 0.04 ***	3.96 ± 0.26 **	23.64 ± 3.54 ***
**analog 3**	7.91 ± 0.46	2.69 ± 0.30	163.13 ± 1.06 ***	0.62 ± 0.03	0.63 ± 0.05	4.86 ± 0.17	40.30 ± 5.57

The data are presented as the mean ± SEM. Statistical analysis was performed using the unpaired t-test.

* P < 0.05

** P < 0.01

*** P < 0.001 vs. the control group treated with saline. FFA–free fatty acids, T Chol–total cholesterol, HOMA–homeostatic assessments treatment

#### Impact of the treatments on the mRNA expression of genes involved in lipid and glucose metabolism and on the mRNA expression of the energy expenditure marker *Ucp1*

**Fig 3:**
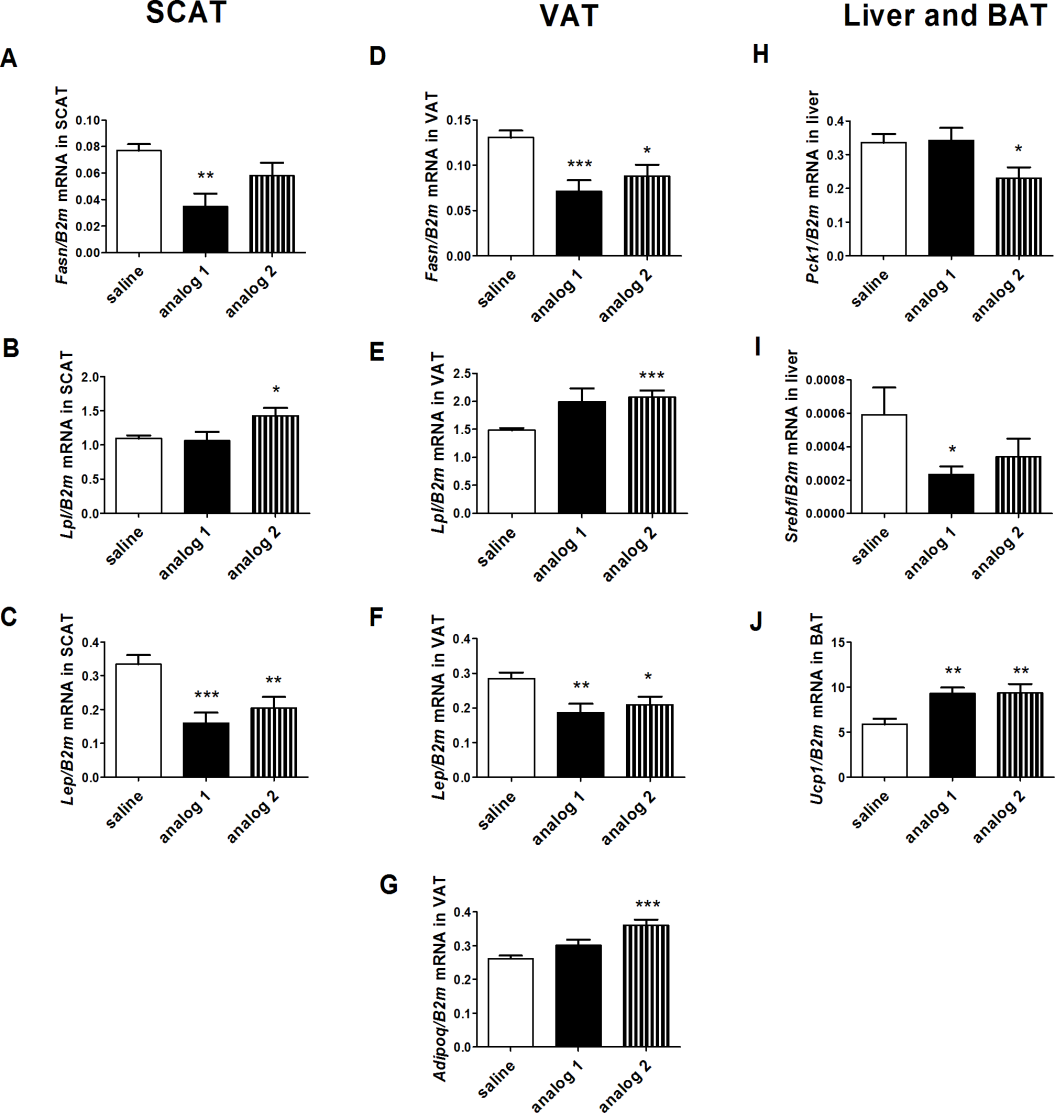
Long-term effects of lipidized PrRP analogs on mRNA expression of genes involved in lipid and glucose metabolism in adipose tissues and livers and on mRNA expression of energy expenditure marker *Ucp1* in DIO mice. **A.** *Fasn* in SCAT, **B.** *Lpl* in SCAT, **C.** *Lep* in SCAT, **D.** *Fasn* in VAT, **E.** *Lpl* in VAT, **F.** *Lep* in VAT, **G.** *Adipoq* in VAT, **H.** *Pck1* in liver, **I.** *Srebf* in liver, **J.** *Ucp1* in BAT. The data are presented as the mean ± SEM. Statistical analysis was performed using the unpaired t-test. * P < 0.05, ** P < 0.01, *** P < 0.001 vs. the HF diet-fed group treated with saline.

Long-term treatment with analog 1 reduced the mRNA expression of Fasn in SCAT and VAT and Srebf in the liver. Treatment with analog 2 decreased the mRNA expression of Fasn in VAT, Pck1 in liver and increased the mRNA expression of Lpl in both SCAT and VAT. Treatment with both of the compounds significantly decreased the mRNA expression of Lep in SCAT and VAT, which was in accordance with the decreased plasma leptin levels and adipose tissue reduction. Expression of adiponectin in VAT tended to increase and was increased after treatment with analogs 1 and 2, respectively. Furthermore, both compounds increased the mRNA expression of Ucp1 in BAT (Fig 3, S1 Data).

### NMR-based metabolomics

**Fig 4:**
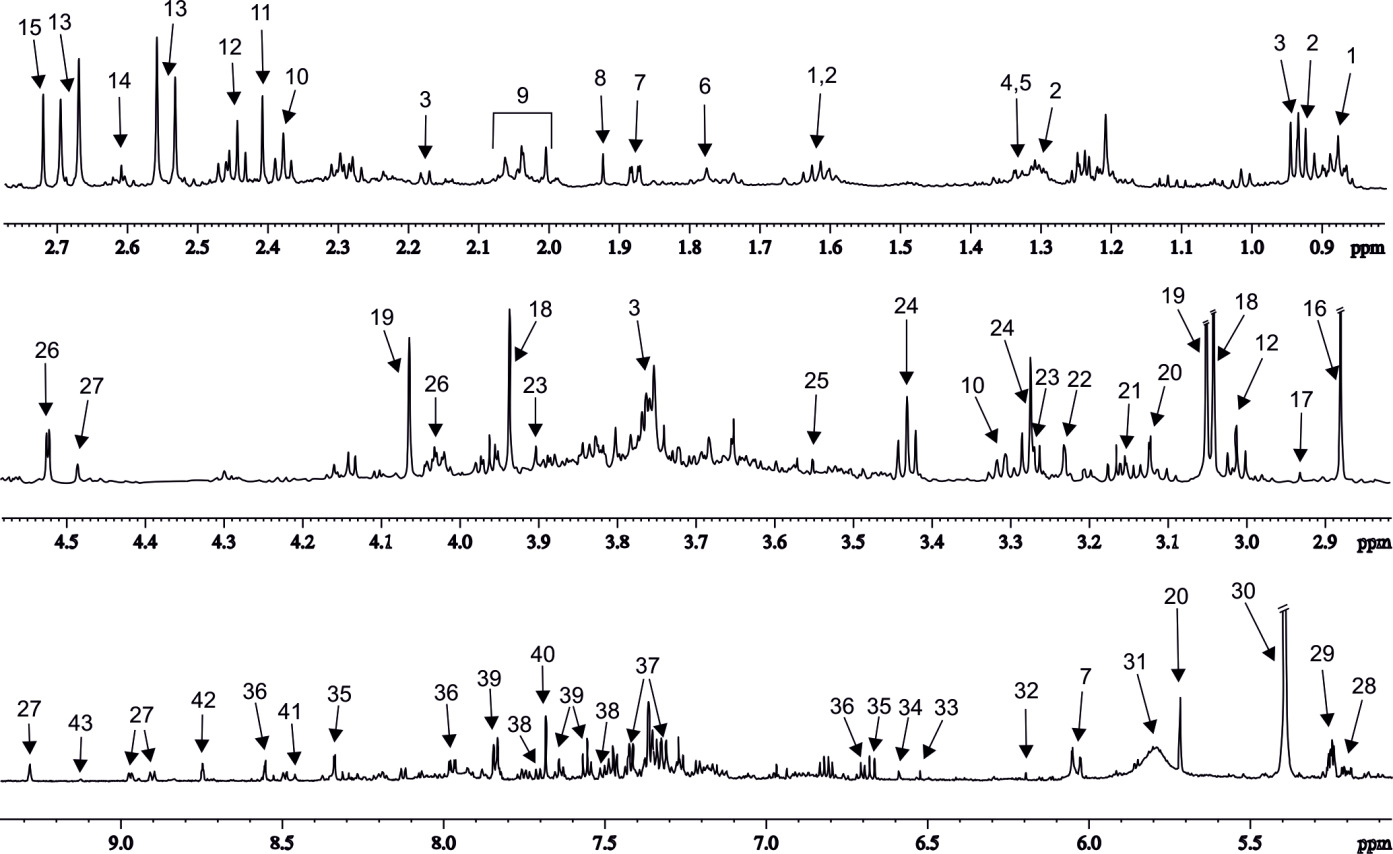
The representative ^1^H NMR spectrum of DIO mouse urine. 1. Hexanoylglycine, 2. 2-Oxovalerate, 3. N-isovalerylglycine, 4. Lactate, 5. 2-Hydroxyisobutyrate, 6. Putrescine, 7. Vinylacetylglycine, 8. Acetate, 9. N-acetyls of aminoacids derivatives, 10. N-carbamoyl-β-alanine, 11. Succinate, 12. 2-Oxoglutarate, 13. Citrate, 14. Methylamine, 15. Dimethylamine, 16. Trimethylamine, 17. N,N-dimethylglycine, 18. Creatine, 19. Creatinine, 20. *cis*-Aconitate, 21. Ethanolamine, 22. Carnitine, 23. Betaine, 24. Taurine, 25. Glycine, 26. Ascorbate, 27. 1-Methylnicotinamide, 28. Glucose, 29. Galactose, 30. Allantoin, 31. Urea, 32. Orotic acid, 33. *trans*-Aconitate, 34. Fumarate, 35. N-methyl-2-pyridone-5-carboxamide, 36. N-methyl-4-pyridone-3-carboxamide, 37. N-phenylacetylglycine, 38. 3-Indoxylsulfate, 39. Hippurate, 40. Guanine, 41. Formate, 42. Nicotinamide N-oxide, 43. Trigonelline.

**Fig 5:**
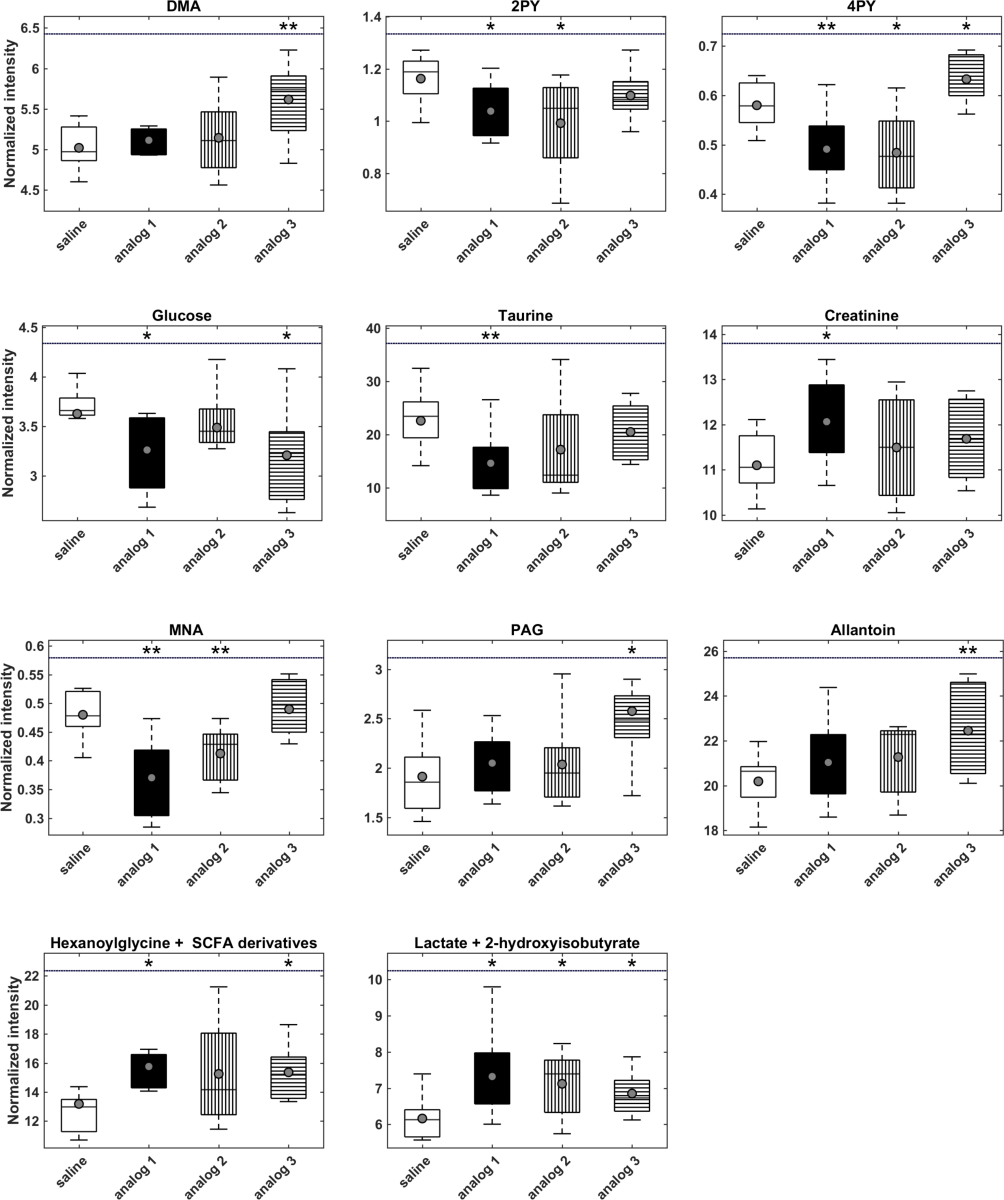
Urinary levels of metabolites significantly changed by the treatment with lipidized PrRP analogs. Statistical analysis was performed using the unpaired t-test. * P < 0.05, ** P < 0.01 vs. the HF diet-fed group treated with saline.

We were able to unambiguously identify 43 metabolites, and the representative signals for each of them are highlighted in Fig 4. A comparison of the urine spectra of the treated mice with the untreated controls on a HF diet revealed significant changes in 12 metabolites including the group of short-chain fatty acids (SCFA) derivatives after a two-week dosing period. The results are summarized using box-plots in Fig 5 and S2 Data.

Treatment with analogs 1 and 2 attenuated the levels of three metabolites of the nicotinamide pathway: 1-methylnicotinamide (MNA), *N*-methyl-2-pyridone-5-carboxamide (2-PY) and *N*-methyl-4-pyridone-3-carboxamide (4-PY). On the contrary, administration of analog 3 raised the concentration of 4-PY. The anti-obesity treatment elevated the intensity of the signals resonating at 0.85–0.91 ppm, and the increase was significant for analogs 1 and 3. The J-resolved experiment showed an overlap of several doublets and triplets in this region, corresponding to hexanoylglycine and various SCFA derivatives (e.g., hydroxy- and keto-derivatives of butyrate, valerate or caproate). A portion of these metabolite resonates were also in the region of 1.58-1.65 ppm where a comparable increase in signal intensity was observed. Unfortunately, the strong signal overlap made the assignment of the metabolites in the above-mentioned regions impossible Therefore, these metabolites were evaluated collectively as SCFA derivatives. All three types of anti-obesity therapy increased the urinary concentrations of the multiplet at 1.34 ppm, which was formed by the overlapping lactate doublet and 2-hydroxyisovalerate singlet. Thus, the contribution of these metabolites to the common bin intensity cannot be independently evaluated. Treatment with analog 1 resulted in a significant increase in the concentration of creatinine. The glucose and taurine levels tended to decrease after therapy. A significant reduction in glucose levels was observed after treatment with analogs 1 and 3, and the taurine concentration was significantly attenuated by administration of analog 1 only. Treatment with analog 3 resulted in growing concentrations of dimethylamine, allantoin and phenylacetylglycine (PAG), and analogs 1 and 2 had no impact on these metabolites.

The significant changes in the metabolites were subsequently correlated with all of the biometric and metabolic parameters, which are listed in Tables 3 and 4 and S1 Data. Table 5, S2 Table and S2 Data summarizes their correlation coefficients with those parameters in which strong correlations were detected: BW; weights of the liver, as well as the subcutaneous, perirenal and total adipose tissues; and plasma levels of insulin, leptin, FFAs, and triglycerides.

**Table 5:** Correlation of significantly changed metabolites with biometric and metabolic parameters.

Metabolite	BW	SCAT	Perir	Total	Liver	Ins	Lep	FFA	TG	HOMA	*Ucp1*
**MNA**	**0.76**	**0.54**	**0.63**	**0.60**	**0.60**	**0.61**	0.46	**0.52**	**0.51**	**0.57**	-0.46
**2-PY**	**0.58**	0.38	**0.52**	0.34	0.48	**0.58**	0.37	0.36	**0.51**	**0.56**	**-0.52**
**4-PY**	**0.63**	0.34	**0.52**	0.48	0.49	**0.53**	0.49	0.43	**0.65**	**0.54**	-0.41
**Taurine**	0.44	0.49	**0.54**	**0.59**	0.22	0.36	**0.58**	0.30	**0.50**	0.49	-0.40
**Hexanoylglycine + SCFAs derivatives**	-0.47	-0.39	**-0.58**	-0.41	-0.37	-0.48	**-0.53**	-0.37	-0.41	**-0.54**	0.27
**Lactate + 2-hydroxyisobutyrate**	-0.48	-0.27	**-0.50**	-0.33	-0.30	-0.46	-0.36	-0.30	-0.22	-0.47	0.06

All correlation coefficients with absolute value ≥ 0.5 and statistical significance (P < 0.05) are printed in bold. Perir–perirenal adipose tissue, Total–total adipose tissue, Ins–insulin, Lep–leptin, TG–triglycerides.

The ellipses in a correlation matrix represent the level of correlation. Perir–perirenal adipose tissue, Total–total adipose tissue, Ins–insulin, Lep–leptin, TG–triglycerides, BW–body weight, SCAT subcutaneous adipose tissue, FFA–free fatty acids, HOMA–homeostatic assessments treatment, *Ucp1* –uncoupling protein 1.

The relevant metabolites formed two subgroups according to the signs of the correlation coefficients: the metabolites of nicotinamide (MNA, 2-PY, 4-PY) and taurine showed all positive correlations; and all of the correlations of the SCFA derivatives, 2-hydroxyisobutyrate and lactate were all negative. MNA was strongly correlated with all nine of the evaluated parameters except plasma leptin, and 2-PY and 4-PY were strongly correlated with BW, perirenal fat weight, and plasma levels of insulin and triglycerides. Taurine was correlated with the concentrations of leptin and triglyceride and with the mass of total and perirenal fat. On the contrary, the SCFA derivatives 2-hydroxyisobutyrate and lactate had strongly negative correlations with the weight of perirenal fat and the leptin concentrations. The other metabolites that were significantly affected by the anti-obesity treatment, e.g., dimethylamine (DMA), allantoin, PAG, glucose, and creatinine, did not show any strong correlations with the acquired biometric or metabolic parameters.

## Discussion

PrRP and its receptor may represent a new promising target for obesity treatment [28]. Our previous studies have demonstrated that the lipidization of PrRP enables its central anorexigenic effects after peripheral administration in both acute and chronic experiments [10, 29]. Data from experimental rodent models also confirmed that the GPR10 and/or NPFF2 receptors are suitable targets for the treatment of obesity [4, 7].

In this study, we explored the biological properties of newly designed analogs of PrRP31 that were palmitoylated through a linker of gamma-glutamic acid or short, modified polyethylene glycol at position 11, as well as an analog with two palmitoyls, one at the N-terminus and one at position 11. The choice of position for lipidization was based on our previous study in which PrRP20 myristoylated at the N-terminus showed a similar biological potency to PrRP31 palmitoylated at the N-terminus [10]. PrRP31 and PrRP20 share an identical C-terminus [30]. PrRP20 was found to be the minimal sequence necessary for the preservation of full *in vivo* activity [31], and the use of palmitic acid for the lipidization of PrRP31 resulted in the most potent analog of the tested series [10]. Therefore, in this study, the amino acid at position 11 of PrRP31 (Lys substituted for the original Arg) was palmitoylated at the secondary amino group through an amide bond, which should result in a biologically active analog. Moreover, the use of a linker between the fatty acid and peptidic chain could enhance the solubility and possibly the potency of these analogs, as shown for the lipidized glucagon-like peptide (GLP-1) analogs [32].

### *In vitro* and *in vivo* studies of the analogs

Two of three novel palmitoylated PrRP31 analogs in this study (analogs 1 and 2) exhibited binding affinities and signaling properties in GPR10-expressing cells that were similar to natural PrRP31. In addition, all of these analogs also showed high binding affinity to the anorexigenic NPFF2 receptor. Thus, *in vitro* experiments confirmed that a single palmitoylation of PrRP31 at position 11 resulted in an affinity for GPR10 that was comparable to those of analogs palmitoylated at the N-terminus [10]. Finally, analogs 1 and 2 showed even higher agonistic potency to GPR10 than natural PrRP31. However, the di-palmitoylated analog 3 exhibited a decrease in binding affinity for and less activation of GPR10 compared to analog 1 and 2, pointing to a lower potency, but its affinity to NPFF2 receptor was comparable to that of analog 1 and 2.

In our previous study, the peripheral administration of myristoylated and palmitoylated PrRP analogs in fasted mice induced strong and long-lasting anorexigenic effects, as well as neuronal activation in the areas of the brain that are involved in food intake regulation [10]. Here, we also demonstrate very significant anorexigenic effects of all three analogs after a single subcutaneous injection to fasted mice. The central mode of action of this effect was again supported by the increase in c-Fos immunoreactivity in the hypothalamic Arc, PVN and DMN nuclei which are involved in food intake regulation [33].

**Fig 6:**
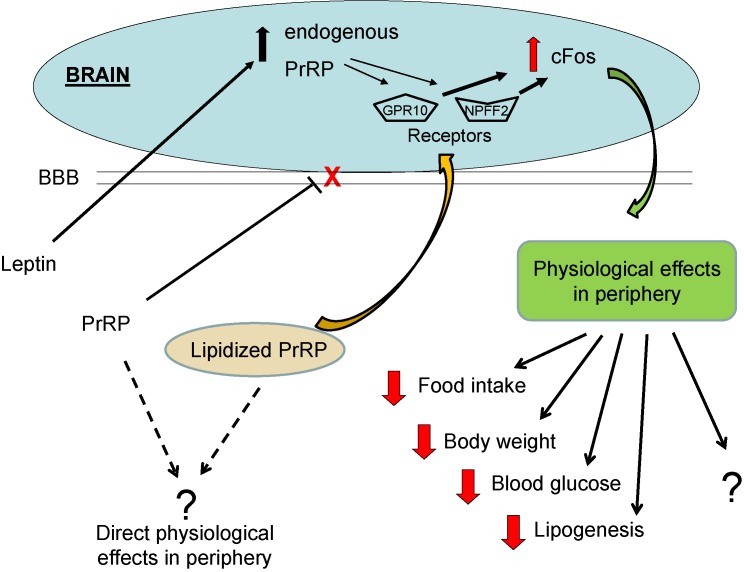
Scheme of proposed mechanism of action of lipidized PrRP analogs - after peripheral injection, actin centrally. BBB blood brain barrier.

We have demonstrated that natural PrRP administered peripherally had no effect on food intake and body weight, but lipidized PrRP had strong anorexigenic effect [10]. In our recent review we suggested that the effect of lipidized PrRP on food intake should be central [28] because of several reasons. Based on an increase in c-Fos immunostaining in Arc, PVN, DMN and NTS, possibly through stimulation of GPR10 and NPFF2 receptors located there [34, 35], we can assume that lipidized PrRP crossed BBB (Fig 6). Moreover, the central neuronal activation of c-Fos after peripheral application of lipidized PrRP is also supported by the selective activation of specific hypothalamic oxytocin and hypocretin neuronal subpopulations [36] both involved in food intake inhibition as well as in energy expenditure. However, we cannot exclude peripheral effects of peripherally administered lipidized PrRP, even we have not observed any indications up to now. Similarly, we did not registered any effects of natural PrRP on locomotor activity and analgesia in tests with mice [10].

The potential anti-obesity and glucose-lowering effects were then investigated by repeated administration of three lipidized PrRP31 analogs in DIO mice, a model of obesity and prediabetes [15]. A previous study has shown that two weeks of subcutaneous administration of palmitoylated-PrRP31 and myristoylated-PrRP20 decreases food intake and body weight, improves metabolic parameters, and attenuates lipogenesis in mice with DIO [10]. To compare this recent study to our previous results, we used an identical experimental design. Analogs 1 and 2 decreased body weight of the DIO mice to a similar extent as the N-palmitoylated PrRP31, i.e. approximately by 12% of the original weight in two weeks. Surprisingly, analog 1 significantly lowered BW despite the fact that food intake after its administration was not significantly lowered. The significant lowering of body fat was confirmed by the decrease in subcutaneous and perirenal fat pad weights and the lowering of leptin levels, as well as by the decrease in liver weight. Moreover, analogs 1 and 2 tended to decrease fasting glucose levels and significantly lowered insulin, HOMA index, free fatty acid, cholesterol and triglyceride levels after two weeks of treatment. The improvements in the above-mentioned metabolic parameters and related HOMA index suggest stronger anti-obesity properties and glucose-lowering potential compared with N-palmitoylated PrRP31 [10]. Finally, administration of analog 3 (the di-palmitoylated analog) tended to decrease BW and improve metabolic parameters, but the results did not reach significance.

The decrease in the adipose tissue mass after treatment with analogs 1 and 2 resulted in a significant attenuation of the mRNA expression of leptin, which is in agreement with the effects observed after administration of N-palmitoylated PrRP31 [10] and with that from previous studies with body weight lowering drugs [37]. Interestingly, analog 1 tended to increase mRNA expression of adiponectin in visceral fat while analog 2 significantly reduced it. Furthermore, the possible lipolytic features of analog 2 were suggested by an increase in mRNA expression of lipoprotein lipase, which is the major enzyme responsible for hydrolysis of triglyceride molecules [38] in adipose tissue. Fatty acid synthase mRNA expression in adipose tissue, which is one of most significant sites of lipogenesis, was significantly reduced after treatment with both PrRP31 analogs. This fact, together with the attenuation of sterol regulatory element-binding protein mRNA expression in the liver, suggests that the effects of the analogs were primarily the result of a decrease in *de novo* lipogenesis, which was similar to the previously described effects of the N-palmitoylated PrRP31 analogs [10].

In addition, treatment with analogs 1 and 2 significantly increased mRNA levels of uncoupling protein 1 (*Ucp-1*) in brown adipose tissue of DIO mice. This result points to a possible increase in energy expenditure and reveals a potential additional mechanism of anti-obesity properties of these analogs. Furthermore, this finding is also in accordance with the knowledge that PrRP receptor knockout mice exhibit decreases in energy expenditure [8].

### NMR-based metabolomics

Two weeks of treatment with analogs 1 and 2 significantly decreased urinary concentrations of MNA and its oxidation products 2-PY and 4-PY compared with the untreated high fat diet-fed group. An increase in these metabolites was previously reported to be associated with obesity in a mouse model of DIO [25], as well as in a model of chemically induced obesity [39] and in genetically obese *db/db* mice [40]. An increase in the urinary levels of MNA metabolites indicates peroxisome proliferation [41, 42], which is related to inflammation, obesity and metabolic syndrome [43, 44]. Based on a comparison of the urinary changes associated with T2DM in mice, rats and humans, MNA and 2-PY have been proposed as suitable biomarkers for T2DM monitoring [40]. Analogously to our recent study using a DIO mouse model [15], we observed an increase in 2-PY and 4-PY in urine from HF diet-fed mice, which was attenuated by oral administration of the anti-diabetic drug vildagliptin alone or in combination with metformin. Similarly, subcutaneous treatment with liraglutide also decreased urinary levels of 2-PY and 4-PY in obese mice of the same DIO mouse model (unpublished results). Thus, the treatment-induced decrease in MNA, 2-PY, and 4-PY observed in our current study is consistent with all previously published results. Interestingly, analog 3 had only a negligible impact on the BW and other biometric and metabolic parameters (see Fig 2 and Tables 3 and 4) reflected in the urinary metabolic profiles. Treatment with analog 3 did not influence the levels of MNA and 2-PY and conversely increased the level of 4-PY.

Administration of analog 1 significantly decreased taurine concentrations compared to urine from untreated HF-diet-fed controls. Similar observations were made in the same DIO mouse model after 2 weeks of subcutaneous liraglutide treatment (unpublished results) and 7 weeks of oral vildagliptine treatment [15]. Elevated urinary excretion of taurine is considered to be an indicator of liver damage [45], which can be induced by inflammation related to lipid β-oxidation and oxidative stress [46]. The depletion of urinary taurine after treatment with PrRP palmitoylated 1 and 2 analogs could therefore indicate an improvement in liver steatosis through inhibition of taurine synthesis.

A statistically significant increase in hexanoylglycine and unassigned acids, collectively referred to as SCFA derivatives, was observed after treatment with analogs 1 and 3, and this finding is in agreement with our previous studies. A reduction in the concentrations of these metabolites was detected in urine from mice with chemically induced obesity [39]. The low levels of SCFA derivatives in urine from mice with DIO increased after anti-diabetic therapy with metformin, vildagliptin and a combination of the two [15], as well as after subcutaneous treatment with liraglutide (unpublished results).

Acylglycines such as hexanoylglycine are formed from glycine and fatty acid residues, which are produced during the β-oxidation process [47]. Conjugation with glycine then facilitates the detoxification of the respective acyl excess from the organism. Therefore, the therapy-induced increase in hexanoylglycine concentrations in urine that was observed in our study may suggest enhanced β-oxidation of fatty acids. The treatment resulted in overproduction of SCFA derivatives generated during BCAA catabolism, which has been repeatedly associated with obesity and the development of T2DM [48]. However, it is important to consider the fasting state at the time of sample collection when discussing the concentration of BCAA catabolites in urine [49].

Treatment with analog 3 elevated the concentrations of DMA, PAG, and allantoin, but administration of analogs 1 and 2 had no impact on these metabolites. Moreover, none of these metabolites correlated with any of the studied biometric and biochemical parameters. Urinary levels of DMA and PAG may be associated with the activity of gut microflora. DMA can be derived from dietary choline, which is broken down to monoamine, DMA and TMA by the gut microflora [50]. The increase in PAG levels may result from increased uptake of precursors produced by gut bacteria [51]. Allantoin, a degradation product of nucleotide metabolism, has been reported to be a marker of oxidative stress. However, it can be produced in mice not only through the oxidation of uric acid by reactive oxygen species but also via enzymatic reactions with urate oxidase [52]. Therefore, the mechanism responsible for the increase in allantoin levels in mice treated with analog 3 is not clear.

## Conclusions

We explored the biological properties of newly designed analogs of PrRP31 that were palmitoylated through a linker of gamma-glutamic acid or a short modified polyethylene glycol at position 11, as well as an analog with two palmitoyls, one at the N-terminus and one at position 11. *In vitro* experiments confirmed that a single palmitoylation of PrRP31 at position 11 with any of used linkers resulted in a high affinity for the GPR10 and NPFF2 receptors, as well as enhanced signaling. A single injection of the novel PrRP31 analogs palmitoylated at position 11 suggests that it has central effects, as shown by the neuronal activation and decrease in food intake observed in mice. Moreover, 2 weeks of repeated administration of these analogs revealed not only significant decrease in body weight but also improvement in multiple metabolic parameters related to obesity and its related diseases.

However, we are aware of differences in possible mechanisms of particular analogs but further studies are needed to reveal it. One possibility would be that the individual potencies of applied PrRP31 analogs multiplied by its different ratio to simultaneously activate GPR10 receptors (localized in PVN and DMN) and NPFF2 receptors (localized in all hypothalamic nuclei involved in food intake regulation except the PVN) may be responsible for different shift in activation of neuronal network pathways accompanied with the food intake suppression and/or energy expenditure. Use of GPR10 and/or NPFF2 receptor knock-out mice would be beneficial.

Furthermore, in this study we confirmed that 1-methylnicotinamide and its oxidation products 2-PY and 4-PY are strongly associated with the development of obesity and T2DM and possibly may serve as markers for efficacy of anti-obesity therapies. Taken together, our data suggest that newly designed palmitoylated analogs of PrRP31 hold promising features with respect to possible use in the treatment of obesity and its related metabolic complications.

## Supporting information

S1 Data Effects of lipidized PrRP analogs in vitro in vivo. (XLSX)

S2 Data Effects of lipidized PrRP analogs—metabolomics. (XLSX)

S1 Table Characteristics of PrRP31 and its analogs. (DOCX)

S2 Table Correlations between metabolites and biometric and metabolic parameters. The ellipses in a correlation matrix represent the level of correlation. Perir–perirenal adipose tissue, Total–total adipose tissue, Ins–insulin, Lep–leptin, TG–triglycerides, BW–body weight, SCAT subcutaneous adipose tissue, FFA–free fatty acids, HOMA–homeostatic assessments treatment, *Ucp1* –uncoupling protein 1.
(DOCX)

## References

[1] Conde-Frieboes K, Thøgersen H, Lau JF, Sensfuss U, Hansen TK, Christensen L, et al Identification and in vivo and in vitro characterization of long acting and melanocortin 4 receptor (MC4-R) selective α-melanocyte-stimulating hormone (α-MSH) analogues. J Med Chem. 2012;55(5):1969–77. doi: 10.1021/jm201489a . 22335602 10.1021/jm201489a

[2] Royalty JE, Konradsen G, Eskerod O, Wulff BS, Hansen BS. Investigation of safety, tolerability, pharmacokinetics, and pharmacodynamics of single and multiple doses of a long-acting α-MSH analog in healthy overweight and obese subjects. J Clin Pharmacol. 2014;54(4):394–404. doi: 10.1002/jcph.211 ; PubMed Central PMCID: PMCPMC4263154. 24166760 10.1002/jcph.211 PMC4263154

[3] Nagelová V, Pirník Z, Železná B, Maletínská L. CART (cocaine- and amphetamine-regulated transcript) peptide specific binding sites in PC12 cells have characteristics of CART peptide receptors. Brain Res. 2014;1547:16–24. doi: 10.1016/j.brainres.2013.12.024 . 24378198 10.1016/j.brainres.2013.12.024

[4] Lawrence C, Celsi F, Brennand J, Luckman S. Alternative role for prolactin-releasing peptide in the regulation of food intake. Nat Neurosci. 2000;3(7):645–6. doi: 10.1038/76597 . 10862694 10.1038/76597

[5] Taylor M, Samson W. The prolactin releasing peptides: RF-amide peptides. Cell Mol Life Sci. 2001;58(9):1206–15. doi: 10.1007/PL00000934 . 11577979 10.1007/PL00000934 PMC11337406

[6] Jarry H, Heuer H, Schomburg L, Bauer K. Prolactin-releasing peptides do not stimulate prolactin release in vivo. Neuroendocrinology. 2000;71(4):262–7. . 10773746 10.1159/000054544

[7] Engstrom M, Brandt A, Wurster S, Savola JM, Panula P. Prolactin releasing peptide has high affinity and efficacy at neuropeptide FF2 receptors. J Pharmacol Exp Ther. 2003;305(3):825–32. doi: 10.1124/jpet.102.047118 . 12606605 10.1124/jpet.102.047118

[8] Bjursell M, Lennerås M, Göransson M, Elmgren A, Bohlooly-Y M. GPR10 deficiency in mice results in altered energy expenditure and obesity. Biochem Biophys Res Commun. 2007;363(3):633–8. doi: 10.1016/j.bbrc.2007.09.016 . 17904108 10.1016/j.bbrc.2007.09.016

[9] Takayanagi Y, Matsumoto H, Nakata M, Mera T, Fukusumi S, Hinuma S, et al Endogenous prolactin-releasing peptide regulates food intake in rodents. J Clin Invest. 2008;118(12):4014–24. doi: 10.1172/JCI34682 ; PubMed Central PMCID: PMCPMC2575834. 19033670 10.1172/JCI34682 PMC2575834

[10] Maletinska L, Nagelova V, Ticha A, Zemenova J, Pirnik Z, Holubova M, et al Novel lipidized analogs of prolactin-releasing peptide have prolonged half-lives and exert anti-obesity effects after peripheral administration. Int J Obes (Lond). 2015;39(6):986–93. doi: 10.1038/ijo.2015.28 . 25771926 10.1038/ijo.2015.28

[11] Prazienkova V, Ticha A, Blechova M, Spolcova A, Zelezna B, Maletinska L. Pharmacological characterization of lipidized analogs of prolactin-releasing peptide with a modified C- terminal aromatic ring. Journal of physiology and pharmacology: an official journal of the Polish Physiological Society. 2016;67(1):121–8. . 27010901

[12] Nicholson JK, Lindon JC, Holmes E. 'Metabonomics': understanding the metabolic responses of living systems to pathophysiological stimuli via multivariate statistical analysis of biological NMR spectroscopic data. Xenobiotica. 1999;29(11):1181–9. doi: 10.1080/004982599238047 . 10598751 10.1080/004982599238047

[13] Holmes E, Wilson ID, Nicholson JK. Metabolic phenotyping in health and disease. Cell. 2008;134(5):714–7. doi: 10.1016/j.cell.2008.08.026 . 18775301 10.1016/j.cell.2008.08.026

[14] Reily MD, Tymiak AA. Metabolomics in the pharmaceutical industry. Drug Discov Today Technol. 2015;13:25–31. doi: 10.1016/j.ddtec.2015.03.001 . 26190680 10.1016/j.ddtec.2015.03.001

[15] Pelantová H, Bugáňová M, Holubová M, Šedivá B, Zemenová J, Sýkora D, et al Urinary metabolomic profiling in mice with diet-induced obesity and type 2 diabetes mellitus after treatment with metformin, vildagliptin and their combination. Mol Cell Endocrinol. 2016;431:88–100. doi: 10.1016/j.mce.2016.05.003 . 27164444 10.1016/j.mce.2016.05.003

[16] Maletínská L, Pýchová M, Holubová M, Blechová M, Demianová Z, Elbert T, et al Characterization of new stable ghrelin analogs with prolonged orexigenic potency. J Pharmacol Exp Ther. 2012;340(3):781–6. doi: 10.1124/jpet.111.185371 . 22182933 10.1124/jpet.111.185371

[17] Motulsky H, Neubig R. Analyzing radioligand binding data. Curr Protoc Neurosci. 2002;Chapter 7:Unit 7.5. doi: 10.1002/0471142301.ns0705s19 . 18428565 10.1002/0471142301.ns0705s19

[18] Maletinska L, Ticha A, Nagelova V, Spolcova A, Blechova M, Elbert T, et al Neuropeptide FF analog RF9 is not an antagonist of NPFF receptor and decreases food intake in mice after its central and peripheral administration. Brain Res. 2013;1498:33–40. doi: 10.1016/j.brainres.2012.12.037 . 23291266 10.1016/j.brainres.2012.12.037

[19] Pirnik Z, Bundzikova J, Holubova M, Pychova M, Fehrentz JA, Martinez J, et al Ghrelin agonists impact on Fos protein expression in brain areas related to food intake regulation in male C57BL/6 mice. Neurochem Int. 2011;59(6):889–95. doi: 10.1016/j.neuint.2011.08.001 . 21843570 10.1016/j.neuint.2011.08.001

[20] Maletínská L, Maixnerová J, Matysková R, Haugvicová R, Pirník Z, Kiss A, et al Synergistic effect of CART (cocaine- and amphetamine-regulated transcript) peptide and cholecystokinin on food intake regulation in lean mice. BMC Neurosci. 2008;9:101 doi: 10.1186/1471-2202-9-101 ; PubMed Central PMCID: PMCPMC2587474. 18939974 10.1186/1471-2202-9-101 PMC2587474

[21] Lansang MC, Williams GH, Carroll JS. Correlation between the glucose clamp technique and the homeostasis model assessment in hypertension. Am J Hypertens. 2001;14(1):51–3. . 11206679 10.1016/s0895-7061(00)01229-2

[22] Monteleone P, Brambilla F, Bortolotti F, Ferraro C, Maj M. Plasma prolactin response to D-fenfluramine is blunted in bulimic patients with frequent binge episodes. Psychol Med. 1998;28(4):975–83. . 9723152 10.1017/s0033291798006904

[23] Cheng Y, Prusoff WH. Relationship between the inhibition constant (K1) and the concentration of inhibitor which causes 50 per cent inhibition (I50) of an enzymatic reaction. Biochem Pharmacol. 1973;22(23):3099–108. . 4202581 10.1016/0006-2952(73)90196-2

[24] Pelantova H, Buganova M, Anyz J, Zelezna B, Maletinska L, Novak D, et al Strategy for NMR metabolomic analysis of urine in mouse models of obesity—from sample collection to interpretation of acquired data. Journal of pharmaceutical and biomedical analysis. 2015;115:225–35. doi: 10.1016/j.jpba.2015.06.036 . 26263053 10.1016/j.jpba.2015.06.036

[25] Boulangé CL, Claus SP, Chou CJ, Collino S, Montoliu I, Kochhar S, et al Early metabolic adaptation in C57BL/6 mice resistant to high fat diet induced weight gain involves an activation of mitochondrial oxidative pathways. J Proteome Res. 2013;12(4):1956–68. doi: 10.1021/pr400051s . 23473242 10.1021/pr400051s

[26] Wishart DS, Jewison T, Guo AC, Wilson M, Knox C, Liu Y, et al HMDB 3.0—The Human Metabolome Database in 2013. Nucleic Acids Res. 2013;41(Database issue):D801–7. doi: 10.1093/nar/gks1065 ; PubMed Central PMCID: PMCPMC3531200. 23161693 10.1093/nar/gks1065 PMC3531200

[27] Maletinska L, Spolcova A, Maixnerova J, Blechova M, Zelezna B. Biological properties of prolactin-releasing peptide analogs with a modified aromatic ring of a C-terminal phenylalanine amide. Peptides. 2011;32(9):1887–92. doi: 10.1016/j.peptides.2011.08.011 . 21872625 10.1016/j.peptides.2011.08.011

[28] Kunes J, Prazienkova V, Popelova A, Mikulaskova B, Zemenova J, Maletinska L. Prolactin-releasing peptide: a new tool for obesity treatment. J Endocrinol. 2016;230(2):R51–8. doi: 10.1530/JOE-16-0046 . 27418033 10.1530/JOE-16-0046

[29] Holubova M, Zemenova J, Mikulaskova B, Panajotova V, Stohr J, Haluzik M, et al Palmitoylated PrRP analog decreases body weight in DIO rats but not in ZDF rats. J Endocrinol. 2016;229(2):85–96. doi: 10.1530/JOE-15-0519 . 26906745 10.1530/JOE-15-0519

[30] Hinuma S, Habata Y, Fujii R, Kawamata Y, Hosoya M, Fukusumi S, et al A prolactin-releasing peptide in the brain. Nature. 1998;393(6682):272–6. doi: 10.1038/30515 . 9607765 10.1038/30515

[31] Maixnerová J, Špolcová A, Pýchová M, Blechová M, Elbert T, Rezáčová M, et al Characterization of prolactin-releasing peptide: binding, signaling and hormone secretion in rodent pituitary cell lines endogenously expressing its receptor. Peptides. 2011;32(4):811–7. doi: 10.1016/j.peptides.2010.12.011 . 21185342 10.1016/j.peptides.2010.12.011

[32] Madsen K, Knudsen LB, Agersoe H, Nielsen PF, Thogersen H, Wilken M, et al Structure-activity and protraction relationship of long-acting glucagon-like peptide-1 derivatives: importance of fatty acid length, polarity, and bulkiness. J Med Chem. 2007;50(24):6126–32. doi: 10.1021/jm070861j . 17975905 10.1021/jm070861j

[33] Fujii R, Fukusumi S, Hosoya M, Kawamata Y, Habata Y, Hinuma S, et al Tissue distribution of prolactin-releasing peptide (PrRP) and its receptor. Regul Pept. 1999;83(1):1–10. . 10498338 10.1016/s0167-0115(99)00028-2

[34] Adachi S, Mochiduki A, Nemoto H, Sun B, Fujiwara K, Matsumoto H, et al Estrogen suppresses the stress response of prolactin-releasing peptide-producing cells. Neurosci Lett. 2005;380(3):311–5. doi: 10.1016/j.neulet.2005.01.064 . 15862908 10.1016/j.neulet.2005.01.064

[35] Ibata Y, Iijima N, Kataoka Y, Kakihara K, Tanaka M, Hosoya M, et al Morphological survey of prolactin-releasing peptide and its receptor with special reference to their functional roles in the brain. Neurosci Res. 2000;38(3):223–30. . 11070188 10.1016/s0168-0102(00)00182-6

[36] Pirnik Z, Zelezna B, Kiss A, Maletinska L. Peripheral administration of palmitoylated prolactin-releasing peptide induces Fos expression in hypothalamic neurons involved in energy homeostasis in NMRI male mice. Brain Res. 2015;1625:151–8. doi: 10.1016/j.brainres.2015.08.042 . 26362395 10.1016/j.brainres.2015.08.042

[37] Ronveaux CC, Tomé D, Raybould HE. Glucagon-like peptide 1 interacts with ghrelin and leptin to regulate glucose metabolism and food intake through vagal afferent neuron signaling. J Nutr. 2015;145(4):672–80. doi: 10.3945/jn.114.206029 ; PubMed Central PMCID: PMCPMC4381768. 25833771 10.3945/jn.114.206029 PMC4381768

[38] Goldberg IJ, Merkel M. Lipoprotein lipase: physiology, biochemistry, and molecular biology. Front Biosci. 2001;6:D388–405. . 11229871 10.2741/goldberg

[39] Pelantova H, Bartova S, Anyz J, Holubova M, Zelezna B, Maletinska L, et al Metabolomic profiling of urinary changes in mice with monosodium glutamate-induced obesity. Analytical and Bioanalytical Chemistry. 2016;408(2):567–78. doi: 10.1007/s00216-015-9133-0 26577083 10.1007/s00216-015-9133-0

[40] Salek RM, Maguire ML, Bentley E, Rubtsov DV, Hough T, Cheeseman M, et al A metabolomic comparison of urinary changes in type 2 diabetes in mouse, rat, and human. Physiol Genomics. 2007;29(2):99–108. doi: 10.1152/physiolgenomics.00194.2006 . 17190852 10.1152/physiolgenomics.00194.2006

[41] Delaney J, Hodson MP, Thakkar H, Connor SC, Sweatman BC, Kenny SP, et al Tryptophan-NAD+ pathway metabolites as putative biomarkers and predictors of peroxisome proliferation. Arch Toxicol. 2005;79(4):208–23. doi: 10.1007/s00204-004-0625-5 . 15838709 10.1007/s00204-004-0625-5

[42] Ringeissen S, Connor SC, Brown HR, Sweatman BC, Hodson MP, Kenny SP, et al Potential urinary and plasma biomarkers of peroxisome proliferation in the rat: identification of N-methylnicotinamide and N-methyl-4-pyridone-3-carboxamide by 1H nuclear magnetic resonance and high performance liquid chromatography. Biomarkers. 2003;8(3–4):240–71. doi: 10.1080/1354750031000149124 . 12944176 10.1080/1354750031000149124

[43] Kersten S. Peroxisome proliferator activated receptors and obesity. Eur J Pharmacol. 2002;440(2–3):223–34. . 12007538 10.1016/s0014-2999(02)01431-0

[44] Stienstra R, Mandard S, Patsouris D, Maass C, Kersten S, Müller M. Peroxisome proliferator-activated receptor alpha protects against obesity-induced hepatic inflammation. Endocrinology. 2007;148(6):2753–63. doi: 10.1210/en.2007-0014 . 17347305 10.1210/en.2007-0014

[45] Waterfield CJ, Turton JA, Scales MD, Timbrell JA. Taurine, a possible urinary marker of liver damage: a study of taurine excretion in carbon tetrachloride-treated rats. Arch Toxicol. 1991;65(7):548–55. . 1685880 10.1007/BF01973715

[46] Sun YJ, Wang HP, Liang YJ, Yang L, Li W, Wu YJ. An NMR-based metabonomic investigation of the subacute effects of melamine in rats. J Proteome Res. 2012;11(4):2544–50. doi: 10.1021/pr2012329 . 22401608 10.1021/pr2012329

[47] Hunt MC, Siponen MI, Alexson SE. The emerging role of acyl-CoA thioesterases and acyltransferases in regulating peroxisomal lipid metabolism. Biochim Biophys Acta. 2012;1822(9):1397–410. doi: 10.1016/j.bbadis.2012.03.009 . 22465940 10.1016/j.bbadis.2012.03.009

[48] Newgard CB, An J, Bain JR, Muehlbauer MJ, Stevens RD, Lien LF, et al A branched-chain amino acid-related metabolic signature that differentiates obese and lean humans and contributes to insulin resistance. Cell Metab. 2009;9(4):311–26. doi: 10.1016/j.cmet.2009.02.002 ; PubMed Central PMCID: PMCPMC3640280. 19356713 10.1016/j.cmet.2009.02.002 PMC3640280

[49] Adams SH. Emerging perspectives on essential amino acid metabolism in obesity and the insulin-resistant state. Adv Nutr. 2011;2(6):445–56. doi: 10.3945/an.111.000737 ; PubMed Central PMCID: PMCPMC3226382. 22332087 10.3945/an.111.000737 PMC3226382

[50] Asatoor AM, Simenhoff ML. The origin of urinary dimethylamine. Biochim Biophys Acta. 1965;111(2):384–92. . 5879475 10.1016/0304-4165(65)90048-6

[51] Delaney J, Neville WA, Swain A, Miles A, Leonard MS, Waterfield CJ. Phenylacetylglycine, a putative biomarker of phospholipidosis: its origins and relevance to phospholipid accumulation using amiodarone treated rats as a model. Biomarkers. 2004;9(3):271–90. doi: 10.1080/13547500400018570 . 15764292 10.1080/13547500400018570

[52] Dryland PA, Love DR, Walker MF, Dommels Y, Butts C, Rowan D, et al Allantoin as A Biomarker of Inflammation in an Inflammatory Bowel Disease Mouse Model: NMR Analysis of Urine. The Open Bioactive Compounds Journal. 2008;1:1–6. Epub 03/06/2008. doi: 10.2174/1874847300801010001

