# Supplementary material for: Impact of novel palmitoylated prolactin-releasing peptide analogs on metabolic changes in mice with diet-induced obesity
Source: PLoS One. 2017 Aug 18;12(8):e0183449. doi: 10.1371/journal.pone.0183449 (PMC5562305; doi:10.1371/journal.pone.0183449)
Supplement: S1 Table — (DOCX) [file pone.0183449.s003.docx]

**S1 Table. Characteristics of PrRP31 and its analogs.**

| Peptide | Molecular weight found | HPLC retention time |
| --- | --- | --- |
| human PrRP31 | 3661.9 | 15.17 |
| analog 1 | 4001.3 | 21.56 |
| analog 2 | 4173.7 | 22.82 |
| analog 3 | 4715.5 | 30.20 |
