# Supplementary material for: Impact of novel palmitoylated prolactin-releasing peptide analogs on metabolic changes in mice with diet-induced obesity
Source: PLoS One. 2017 Aug 18;12(8):e0183449. doi: 10.1371/journal.pone.0183449 (PMC5562305; doi:10.1371/journal.pone.0183449)
Supplement: S2 Table — The ellipses in a correlation matrix represent the level of correlation. Perir–perirenal adipose tissue, Total–total adipose tissue, Ins–insulin, Lep–leptin, TG–triglycerides, BW–body weight, SCAT subcutaneous adipose tissue, FFA–free fatty acids, HOMA–homeostatic assessments treatment, Ucp1 –uncoupling protein 1. (DOCX) [file pone.0183449.s004.docx]

**S2 Table. Correlations between metabolites and biometric and metabolic parameters.**

The ellipses in a correlation matrix represent the level of correlation. Perir – perirenal adipose tissue, Total – total adipose tissue, Ins – insulin, Lep – leptin, TG – triglycerides, BW – body weight, SCAT subcutaneous adipose tissue, FFA – free fatty acids, HOMA – homeostatic assessments treatment, *Ucp1* – uncoupling protein 1.
